# Obituary

**Published:** 1911-12-23

**Authors:** 


### Obituary.

Dr. George Arthur Slack, of Uffculme, Devon, has died at the age of sixty-four. He qualified from Guy's Hospital in 1886, as member of the Royal College of Surgeons.

Dr. William Wilfrid Webb, formerly major in the Indian Medical Service, has died at his residence at Exeter. Major Webb was a student at Charing Cross Hospital, where he held the valuable appointment of Medical Registrar as well as demonstratorships in Anatomy and Physiology. He qualified in 1878, but continued his studies at the University of Aberdeen, graduating there in 1881 and taking the M.D. in 1894. He was at one time Superintendent of Gaols and Dispensaries for the Meywar and Bikanir States, and was guardian to the Maharajah of Bikanir. At Netley he was curator of the Natural History Museum of the Royal Victoria Hospital, and carried off the Sir Joseph Fayrer prize for pathology and the Martin Memorial Gold Medal in 1883. He was highly successful as a student in gaining prizes, and contributed numerous technical articles to the medical Press, besides writing a guide for candidates for commissions in the Indian Medical Service, a manual of vaccination, and a work on the currencies of the Hindu States of Rajputana.

Dr. John W. Martin, of Sheffield, has died at the age of sixty-five. He was one of the most prominent medical practitioners in Sheffield, and had been twice President of the North of England Obstetrical and Gynæcological Society, and was an ex-President of the local Medico-Chirurgical Society. His medical studies were pursued in Ireland, in Edinburgh, and in Paris; he qualified M.D. and M.Ch. of the Royal University of Ireland in 1868. Dr. Martin began practice in Sheffield in 1879. His contributions to medical literature were numerous, and chiefly on gynæcological subjects. He was formerly lecturer at the Sheffield University on Midwifery and the Diseases of Women. Dr. Martin took a great interest in the teaching of spelling, and was an advocate of the phonetic system; he published a work entitled "The Gordian Knot Cut, or Phonetic Spelling as an Aid to National Education."

Dr. Charles Francis Steele has died under tragic circumstances at the Smedley Hydro, Matlock, at the early age of thirty-nine. He appears to have taken an overdose of veronal, having been suffering from insomnia following influenza. Dr. Steele was very well known in the hydropathic world; he was at the time of his death medical superintendent at the Bridge of Allan Hydro, Stirlingshire, and was formerly resident physician at Smedley's Hydro at Matlock, whither he had gone lately for treatment for himself. He was a student of University College, London, and qualified in 1896, taking the M.D. Lond. in 1901. He held the appointments of Casualty Officer at the East London Children's Hospital, Shadwell, and assistant to the out-patient department at University College Hospital before associating himself with work in hydropathic establishments, where he achieved a notable success.

### A VARIED MEDICAL CAREER.

Dr. Kenneth MacDonald O'Callaghan, B.A. L.F.P.S., and L.M. (Glasgow), and L.S.A. (London) has died at Birkenhead. Dr. O'Callaghan was educated at Dublin University and Queen's University, Ireland, and took his medical degrees in Scotland. The greater part of Dr. O'Callaghan's service was military—this extending over more than thirty years, and before he retired with the rank of Major in the Royal Army Medical Corps, he had been the recipient of seven medals and clasps. He served through the Zulu war, 1879, with Lord Chelmsford's force, and was present at the battle of Ulundi. In India, later, he was attached to the 17th Lancers and the 4th Dragoon Guards. His honourable acquaintance with Freemasonry in India led to his being appointed in 1884 Grand Registrar of the Bombay District. At Poona he was staff surgeon to the suite of the Duke and Duchess of Connaught. After leaving the Army the doctor held a number of important posts. As ship's doctor he served on the White Star, Elder Dempster, Red Cross, and Leyland Lines. He was at various times surgeon to the coastguards, medical officer of health, and public vaccinator, and medical referee to the Prudential, and surgeon to the Stevens's Hospital, Dublin, and to the South Infirmary, Cork. He was on one occasion the guide and interpreter of a British expedition in India. For about ten years he has been a familiar figure in Birkenhead, and was very popular amongst the poor.

### IN MEMORY OF SIR HENRY HARBEN.

The Hampstead General Hospital has received a donation of £51 4s. 4d., being the balance of money subscribed by the chief office-staff of the Prudential Assurance Company for a floral wreath sent to the funeral service of the late Sir Henry Harben, who was president of the assurance company and also president of the hospital.

